# Radiotherapy combined with gefitinib for patients with locally advanced non-small cell lung cancer who are unfit for surgery or concurrent chemoradiotherapy: a phase II clinical trial

**DOI:** 10.1186/s13014-020-01596-2

**Published:** 2020-06-20

**Authors:** Zhixue Fu, Xu Yang, Wenqing Wang, Lei Deng, Tao Zhang, Nan Bi, Xiaozhen Wang, Dongfu Chen, Zongmei Zhou, Luhua Wang, Jun Liang

**Affiliations:** Department of Radiation Oncology, National Cancer Center/National Clinical Research Center for Cancer/Cancer Hospital, Chinese Academy of Medical Sciences and Peking Union Medical College, Beijing, 10021 China

**Keywords:** Non-small cell lung cancer, Radiotherapy, Molecular targeted therapy, Gefitinib

## Abstract

**Background:**

The objectives of this study were to determine the objective effective response rate, survival, and safety of radiotherapy combined with gefitinib in patients with locally advanced non-small cell lung cancer (NSCLC) who were unfit for surgery or concurrent chemoradiotherapy.

**Methods:**

The patients with the locally advanced NSCLC who were unfit to receive surgery or concurrent chemoradiotherapy, received thoracic intensity-modulated radiotherapy (IMRT) combined with gefitinib 250 mg daily.

**Results:**

29 patients were enrolled between July 2014 and March 2017. 28 patients was in the analysis. Of the 28 patients, 21 (75.0%) experienced a partial response, 5 (17.9%) had stable disease, and 2 (7.1%) experienced progression of disease. The objective response rate was 75.0%, and the disease control rate was 92.9%. The median follow-up time was 51 months. The disease progression showed in 25 (89.3%) patients, including local progression in 19 (67.9%) and distant metastasis in 16 (57.1%). The median overall survival and progression-free survival time (PFS) were 26 and 11 months, respectively. The 3-, 4-, 5-year survival rates were 39.0, 30.1 and 30.1%, respectively. The 3-, 4-, 5-year PFS rates were 14.3, 9.5 and 9.5%. Two patients developed grade 3 acute adverse events. Seven patients developed grade 2 acute irradiation pneumonitis, and there was no grade 3 acute irradiation pneumonitis.

**Conclusions:**

For patients with locally advanced NSCLC who are not eligible for surgery or concurrent chemoradiotherapy, IMRT combined with gefitinib can improve the objective effective rate and is generally well-tolerated.

## Background

Lung cancer is a malignant tumor with one of the highest morbidity and mortality rates in the world, and non-small cell lung cancer (NSCLC) accounts for approximately 85% of lung cancers [1]. Approximately 30% of NSCLC are at a locally advanced stage at the time of diagnosis. Concurrent chemoradiotherapy (CRT) plus Durvalumab is the standard treatment for unresectable locally advanced NSCLC; however, not all patients with unresectable stage III NSCLC are able to tolerate concurrent CRT. Previous clinical trials have shown that among patients treated with concurrent CRT, the incidence of grade 3 and more irradiation pneumonitis and esophagitis ranged from 0 to 18% and 3.4–32%, respectively [2–11]. The PACIFC study [12], durvalumb after chemoradiotherapy in stage III NSCLC, showed that a total of 30.5% of the patients in the durvalumab and 26.1% of those in the placebo group had grade 3 and more adverse events.

Among patients who are unable to tolerate concurrent CRT, the conventional treatment regimens are sequential chemotherapy and radiotherapy, or radiotherapy only, and the median overall survival (OS) is 11–16 months [2–9].

Coupled with further development of lung cancer genomics, epidermal growth factor receptor (EGFR) tyrosine kinase inhibitors (TKIs) provide an effective treatment for patients with the advanced lung adenocarcinoma. There is currently a lot of interest in targeted therapy, which has a higher efficiency and a lower toxicity.

Several trials have shown that EGFR inhibitors have a radiotherapy sensitization effect when combined with radiotherapy. The Cancer and Leukemia Group B (CALGB30106) trial [13], which evaluated the feasibility of poor-risk patients receiving radiotherapy and gefitinib (Group A) and those with a more favorable risk profile receiving concurrent CRT and gefitinib (Group B), showed that Group A had a longer MST than Group B. In RTOG 0972, a phase II clinical trial of induction chemotherapy followed by thoracic radiotherapy (TRT) and erlotinib in 75 poor-risk patients with stage III NSCLC, showed the median OS and progression-free survival time (PFS) were 17 and 11 months, respectively, and the 1-year overall survival (OS) was 75% [14].

These trials demonstrate that radiotherapy combined with targeted therapy may be a potentially beneficial treatment for patients with locally advanced NSCLC who are unable to tolerate concurrent CRT. We conducted a phase II trial to evaluate the effectiveness of radiotherapy combined with gefitinib in patients with locally advanced NSCLC who were unfit for surgery or concurrent CRT. The primary objective of this study was to determine the objective response rate. The secondary objectives included assessing the OS, the median OS, PFS, and the safety of radiotherapy combined with gefitinib.

## Patients and methods

### Patient selection

The inclusion criteria were: (1) histologically or cytologically confirmed unresectable stage III NSCLC; (2) no thoracic radiotherapy history; (3) tumor objectively measured; (4) age ≥ 18 years; (5) Karnofsky Performance Status ≥70; (6) predicted survival ≥3 months; (7) without severe disease of the vital organs; (8) provision of written informed consent.

The exclusion criteria were: (1) neurological disease; (2) history of recent treatment for another malignant tumor; (3) previous participation in clinical trials of other new drugs; and (4) previous targeted therapy.

The unresectable stage IIIA and IIIB NSCLC mainly refers to the following image or lymph node pathological evidence: 1. The more ipsilateral mediastinal lymph nodes merging into a great lump or multi-region metastasis (IIIA: T1–3 N2 or IIIB: T4N2); 2. The contralateral hilar,mediastinal lymph nodes; the ipsilateral /contralateral scalene, supraclavicular lymph node metastasis (IIIB: T1- N3); 3. Lesions invades into the heart, the aorta and esophagus (IIIB: T4N0–1).

The patients of NSCLC, unfitting for concurrent chemoradiotherapy, refer to the following situations: the patients refuse the concurrent chemoradiotherapy; the patients have so much basic disease that they can’t toletate the concurrent CRT.

### Treatment

Patients were treated with a combination of radiotherapy and gefitinib:

- **Radiotherapy:** Photon beams were applied with an intensity of 6-MV. The gross tumor volume (GTV) was defined as the volume of the primary disease as well as any involved regional lymph nodes (the short axis of at least 1 cm on computed tomography scan). The clinical target volume (CTV) included the primary tumor plus a margin of 6–8 mm, elective nodal regions including ipsilateral hilar, paratracheal, and subcarinal nodal regions were also included in the CTV. The planning target volume (PTV) included the CTV plus a margin of 5 mm in 3 dimensions. The prescription dose was 95% PTV 60–66 Gy/2 Gy/30–33 f.
- **Gefitinib:** An oral dose of 250 mg was administered daily, starting on Day 1. If the treatment was effective, it was continued after radiotherapy had been completed.

### Response and toxicity criteria

The Response Evaluation Criteria in Solid Tumors (RECIST) guideline version 1.0 was used to evaluate the short-term effectiveness. The National Cancer Institute (NCI) Common Terminology Criteria for Adverse Events (CTCAE) version 3.0 was used to evaluate the toxicity.

### Study design

The study was a single-arm, phase II open-label clinical trial.

### Calculation of sample size

In the JCOG0301 trial [15], the objective effective rate of concurrent CRT was 51.5%. We expected that the objective effective rate of TRT with gefitinib would be 68%. According to the sample size calculations, at least 27 patients would be required. The target sample size was increased by 10% to 30 patient to allow for participant withdrawals.

### Statistical analysis

The statistical analysis was performed using SPSS version 20.0 (IBM Corp, Armonk, NY, USA). Kaplan-Meier plots were used for survival analysis. Log-rank tests were used to assess the significance of associations in the univariate analysis. Cox regression was using to assess factors associated with survival in the multivariate analysis. Results with *p* < 0.05 were considered statistically significant.

## Results

### Patient characteristics

From July 2014 to March 2017, 29 patients were enrolled, of which 28 were included in the analysis. One patient refused to continue radiotherapy because of the fever, so the patient was excluded from the analysis. The median age of the participants was 63 years. Of the participants, 7 (25.0%) and 21 (75.0%) were diagnosed with stages III A and III B NSCLC, respectively. Seventeen participants (60.7%) had previously received platinum-based induction chemotherapy (Table 1).

**Table 1:** Patients’ characteristics

Characteristics	N(%)
**Sex**	
Male	24 (85.7)
Female	4 (14.3)
**Age**	
Median	63
Range	42–77
**Smoking index**	
0	7 (25.0)
1–400	1 (3.6)
≥ 400	20 (71.4)
**KPS status**	
70	2 (7.1)
80	16 (57.1)
90	9 (32.1)
100	1 (3.6)
**Weight loss within 6 months before the treatment**	
0	19 (67.9)
<5%	4 (14.3)
≥ 5%	5 (17.9)
**T stage**	
T1	1 (3.6)
T2	10 (35.7)
T3	9 (32.1)
T4	8 (28.6)
**N stage**	
N1	3 (10.7)
N2	9 (32.1)
N3	16 (57.1)
**Clinical stage**	
IIIA	7 (25.0)
IIIB	21 (75.0)
**Histological type**	
• Squamous cell carcinoma	15 (53.6)
Adenocarcinoma	12 (42.9)
Neuroendocrine carcinoma	1 (3.6)
**Inducing chemotherapy before TRT**	
Yes	17 (60.7)
No	11 (39.3)

### Treatment regimens

Of the 28 participants, 21 received intensity-modulated radiotherapy and 7 received volumetric-modulated arc therapy. Twenty eight patients completed TRT with a dose of 54–60Gy.Twenty three patients accepted TRT with 60Gy. Three patients completed TRT with 58Gy. Because the CTV of the 3 patients is too large and the V20 of double lungs is beyond 28%, the total dose reduced to 58Gy. Two patients accepted TRT with 54Gy, because they suffered from grade 2 radiation pneumonia.

### Response to treatment and toxicity

Of the 28 patients, 21 (75.0%) experienced a partial response (PR), 5 (17.9%) had stable disease (SD), and 2 (7.1%) experienced progression of disease (PD). None of the patients experienced a complete response (CR). The objective response rate (CR + PR) was 75.0%, and the disease control rate (CR + PR + SD) was 92.9%. Twenty-five participants (89.3%) experienced a relapse: 19 (67.9%) experienced a local relapse, and 16 (57.1%) experienced a distant relapse, including 10 participants (35.7%) who experienced both a local and a distant relapse.

Overall, the toxicity results demonstrated the feasibility of this approach (Table 2). One patient (3.6%) experienced Grade 3 liver dysfunction, esophagitis, and diarrhea, respectively. Seven participants (25.0%) experienced grade 2 pneumonitis and there was no grade 3 acute irradiation pneumonitis.

**Table 2:** Acute adverse events

Toxicity	Toxicity Grade			
	1 N(%)	2 N(%)	3 N(%)	4 N(%)
**Skin**				
Rash	9 (32.1)	0	0	0
Radiation dermatitis	18 (64.3)	0	0	0
**Hematologic toxicity**				
Anemia	9 (32.1)	0	0	0
Leukopenia	9 (32.1)	0	0	0
Thrombocytopenia	0	0	0	0
**Gastrointestinal toxicity**				
Nausea	7 (25.0)	3 (10.7)	0	0
Vomiting	1 (3.6)	1 (3.6)	0	0
Diarrhea	1 (3.6)	0	1 (3.6)	0
**Esophagitis**	9 (32.1)	9 (32.1)	1 (3.6)	0
**Hypohepatia**	8 (28.6)	0	1 (3.6)	
**Renal function**	6 (21.4)	0	0	0
**Pneumonitis**	2 (7.1)	7 (25.0)	0	0

### Survival

**Fig. 1:**
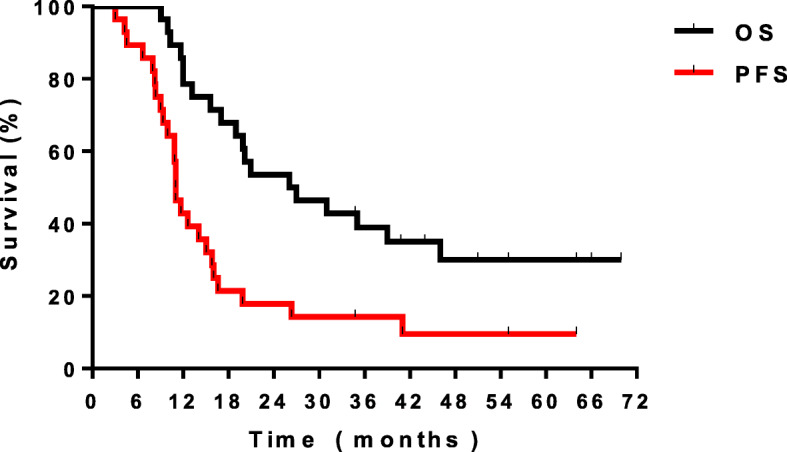
The O S and P FS curves of the w hole group

Follow-up was censored on 15 December 2019. The 7median follow-up time was 51 months. The median PFS was 11 months, and the MST was 26 months. The 3-, 4- and 5-year survival rates were 39.0, 30.1 and 30.1%, respectively. The 3-, 4- and 5-year PFS rates were 14.3, 9.5 and 9.5%, respectively. (Fig. 1).

Univariate analysis showed that only disease stage was associated with OS (*p* = 0.043, 95%CI 11.998–40.109), and with PFS (*p* = 0.000, 95%CI 9.899–12.114).

The median OS of participants with stages III A and III B disease were 12 and 35 months, respectively. Participants with stage III B disease had a significantly better OS and PFS than patients with stage III A disease.

### Epidermal growth factor receptor mutation

Molecular data were available for 13 participants. The EGFR-activating mutation was detected in 6 of the 13 participants (46.2%). The 6 participants with the EGFR-activating mutation had a significantly better the median OS (39 months) than the 20 participants with the EGFR wild-type or non-adenocarcinoma (20 months) without significant difference.

## Discussion

In this trial, the PR rate and the objective effective response rate were both 75.0%, and the disease control rate was 92.9%, and no participants experienced a CR. In previous clinical trials of concurrent CRT the CR rate was 9–28%, the PR rate was 28–81%, and the objective effective response rate was 43–85% [2–11, 15]. Among participants in previous studies who received radiotherapy only, or sequential chemotherapy and radiotherapy, the CR rate was 1.3–30%, the PR rate was 30–65%, and the objective effective response rate was 39–66%. Although none of the participants in our study experienced a CR, the PR rate and the objective effective response rate were comparable to those of previous studies.

The reason for the absence of any participants with a CR in this study may be attributable to: the high proportion of participants with stage III B disease (75%); high PTV (64.3% had a PTV volume > 450 ml); the relatively small proportion of participants with adenocarcinoma (42.9%). The relatively high PR rate and objective effective response rate compared to previous studies may be attributable to the higher rates of completion of radiotherapy (82.1%) and targeted therapy (85.7%).

In the CALB30106 trial [13] the high-risk group (radiotherapy and gefitinib) had a median OS of 19.0 months and median PFS of 13.4 months; while the low-risk group (concurrent CRT and gefitinib) had a median OS of 13 months and a median PFS of 9.2 months. The survival time of high-risk patients who received sequential CRT with gefitinib is promising. Spanish researchers [16] studied 90 patients with locally advanced NSCLC. The median OS was 11.4 months among those who received radiotherapy only, and 8.9 months among those who received radiotherapy with erlotinib, and the median PFS was 15.3 months and 12.9 months, respectively. Wang et al. reported that EGFR-TKI concurrent with thoracic radiotherapy in treating stage IIIB/IV NSCLC had local control rate of 96% for thoracic tumor and 1-year PFS rate of 42% [17]. Zheng et al. reported that the 1-year PFS rate of 57.1%, the median PFS 13 months and the median time to progression of irradiated lesion 20.5 months in TIK combined with radiotherapy as first line treatment for patients with stage IV NSCLC harboring EGFR active mutations [18]. In the RTOG 9410 trial [4], the median OS was 17 months among participants who received 60 Gy of CRT, and 15.6 months among those who received 70 Gy of CRT. Compared with radiotherapy only and concurrent CRT, the survival of participants who received radiotherapy combined with targeted therapy is comparable to that of the participants in our study.

In our study, the OS and PFS of participants with stage III B disease was higher than that of participants with stage III A disease. Possible reasons for this paradoxical result include: (1) participants with stage III A disease had more risk factors than those with III B disease including older age(≥65 year; III A 5/7 (71.4%);III B 8/21 (38.1%);), greater weight loss(6 months before radiotherapy: weight loss≥5%;III A 3/7 (42.9%);III B 2/21 (9.5%);), more comorbidities (including high blood pressure, diabetes, heart disease and other disease; III A 6/7 (85.7%);III B16/21 (76.2%);), less healthy lifestyles (smoking index ≥400;III A 6/7 (85.7%);III B 14/21 (66.7%);), and larger primary tumors (maximum diameter before radiotherapy≥4 cm;III A 5/7 (71.4%);III B 10/21 (47.6%);); (2) participants with stage III B disease were more likely to have adenocarcinoma and EGFR mutations than those with stage III A disease (adenocarcinoma; III A 2/7 (28.6%);III B 10/21 (47.6%);) (EGFR mutations; III A 1/7 (14.3%);III B 5/21 (23.8%);); (3) participants with stage III B disease received more induction chemotherapy (III A 3/7 (42.9%);III B 14/21 (66.7%);); (4) participants with stage III A disease suffered more acute toxicities (Grade 3 esophagitis; III A 1/7 (14.3%);III B 0/21 (0);) (Grade 3 diarrhea; III A 1/7 (14.3%);III B 0/21 (0);).

In our study, participants with the EGFR-activating mutation had a better the median OS than those with the EGFR wild-type and those with non-adeno carcinomatous tumors. This result suggests that there is an association between the EGFR mutation state and response to targeted therapy. For NSCLC, previous studies have shown that approximately 80% of patients with squamous cell carcinoma and 65% of patients with adenocarcinomas have overexpression of EGFR protein, and the overexpression state is an important factor leading to radiation resistance [19, 20]. Meta-analysis conducted in 2002 suggested that a high EGFR expression could be related to the prognosis of NSCLC [21]. However, the CALGB 30106 trial [13] did not find an association between the presence of EGFR mutations and the prognosis of NSCLC. Although the relationship between EFGR-mutation and prognosis in advanced lung adenocarcinoma was relatively clear, the heterogeneity was relatively large among participants with locally advanced lung adenocarcinoma who were able to receive radiotherapy and chemotherapy. For locally advanced lung cancer, there are no prospective studies that have assessed whether individuals with EGFR-activating mutations could benefit from targeted therapy as the first-line treatment. Our study suggests that radiotherapy combined with gefitinib could improve the survival of patients with locally advanced EGFR-activating mutation lung adenocarcinoma.

In this study, the majority of acute adverse events (92.9%) were grades 1 and 2; only 7.1% of participants experienced a grade 3 acute adverse event, and no participants experienced a grade 4 acute adverse event. These results indicate that radiotherapy combined with gefitinib was well tolerated.

Irradiation pneumonia is an important adverse event among patients treated with TRT. In our study, the incidence of grade 2 acute irradiation pneumonitis was 25.0%, but there were no cases of more than grade 3 acute irradiation pneumonitis. Compared with concurrent CRT (which has a reported rate of grade 3 acute irradiation pneumonitis of 0–18%) [2–11], targeted therapy combined with radiotherapy did not significantly increase the incidence of related adverse events. Although irradiation pneumonia did not cause any treatment-related deaths among participants in our study, the pulmonary toxicity associated with EGFR-TKIs is a cause for concern. Zheng et al. reported that the most common grade 3 adverse events were radiation pneumonitis (20%) and rash (10%) in TIK combined with radiotherapy as first line treatment for patients with stage IV NSCLC harboring EGFR active mutations [18]. A study [22] of the relationship between radiotherapy combined with erlotinib and acute irradiation pneumonitis among 24 patients with NSCLC, suggested that 9 patients (37.5%) experienced greater than grade 2 acute irradiation pneumonitis. Other studies have shown that erlotinib can damage the lung stroma [23, 24], and erlotinib combined with TRT may increase the occurrence of acute irradiation pneumonitis. Some small sample studies [25, 26] also reported there was a higher incidence of acute irradiation pneumonitis among patients treated with erlotinib combined with TRT.

This trial had several limitations. First, some patients received induction chemotherapy, but the induction chemotherapy regimens and chemotherapy cycles were not standardized. Second, this study was a single-arm, phase II clinical trial with no control group. Third, the sample was small.

## Conclusions

For patients with locally advanced NSCLC who couldn’t receive surgery or concurrent chemoradiotherapy, TRT combined with gefitinib could improve the objective effective rate and be tolerated well.

## Data Availability

**T**he data and materials of this study are available from the corresponding author on reasonable request.

## Abbreviations

CR: Complete response
CRT: Chemoradiotherapy;
CTCAE: Common terminology criteria for adverse events
CTV: Clinical target volume
EGFR: Epidermal growth factor receptor
IMRT: Intensity-modulated radiotherapy
MST: Median survival time
NCI: National cancer institute
NSCLC: non-small cell lung cancer
OS: Overall survival
PD: Progressive disease
PFS: Progression-free survival
PR: Partial response
PTV: Planning target volume
RECIST: Response evaluation criteria in solid tumors
SD: Stable disease
TKI: Tyrosine kinase inhibitor
TRT: Thoracic radiotherapy
